# Looking Behind the Curtain: Identifying Factors Contributing to Changes on Care Outcomes During a Large Commercial EHR Implementation

**DOI:** 10.5334/egems.269

**Published:** 2019-05-06

**Authors:** Tiago K. Colicchio, Damian Borbolla, Vanessa D. Colicchio, Debra L. Scammon, Guilherme Del Fiol, Julio C. Facelli, Watson A. Bowes, Scott P. Narus

**Affiliations:** 1 University of Alabama at Birmingham, US; 2 University of Utah, US; 3 Intermountain Healthcare, US

**Keywords:** Electronic Health Records, Adoption, Medical Informatics Applications, Outcome Assessment, Mixed-method

## Abstract

**Objective:**

To identify factors contributing to changes on quality, productivity, and safety outcomes during a large commercial electronic health record (EHR) implementation and to guide future research.

**Methods:**

We conducted a mixed-methods study assessing the impact of a commercial EHR implementation. The method consisted of a quantitative longitudinal evaluation followed by qualitative semi-structured, in-depth interviews with clinical employees from the same implementation. Fourteen interviews were recorded and transcribed. Three authors independently coded interview narratives and via consensus identified factors contributing to changes on 15 outcomes of quality, productivity, and safety.

**Results:**

We identified 14 factors that potentially affected the outcomes previously monitored. Our findings demonstrate that several factors related to the implementation (e.g., incomplete data migration), partially related (e.g., intentional decrease in volume of work), and not related (e.g., health insurance changes) may affect outcomes in different ways.

**Discussion:**

This is the first study to investigate factors contributing to changes on a broad set of quality, productivity, and safety outcomes during an EHR implementation guided by the results of a large longitudinal evaluation. The diversity of factors identified indicates that the need for organizational adaptation to take full advantage of new technologies is as important for health care as it is for other services sectors.

**Conclusions:**

We recommend continuous identification and monitoring of these factors in future evaluations to hopefully increase our understanding of the full impact of health information technology interventions.

## Introduction

Electronic health record (EHR) systems adoption in the United States has increased to rates never observed before [1], and, as a result, the literature evaluating the impact of health information technology (HIT) interventions on quality, productivity, and safety outcomes has also increased [2]. However, previous studies have produced mixed results, leaving unanswered questions as to the impact of HIT interventions [2]. The lack of consistent evidence has been attributed primarily to insufficient descriptions of study settings and interventions; the use of a narrow set of study-specific measurements; and weak research designs that do not consider the longitudinal effects introduced by HIT interventions [2]. Previous studies suggest that HIT is seldom objectively measured, and that claims regarding its efficacy are often based on self-promotion and not on scientific evidence [3]. It has been estimated that without improved research methods, around 100 hypotheses per year will continue to be tested without providing any valuable knowledge [4].

Studies from other service sectors such as retail, transportation, and finance, demonstrate that information technology (IT) adoption tends to produce positive outcomes only when accompanied by complementary changes or investments (e.g., proper training, upgrading IT infrastructure, adapting workflows) necessary to take full advantage of new technologies [5]. Such factors have not been explored in evaluations of IT adoption in the health care industry and deserve further attention from the broader medical and informatics communities [6].

In previous work, we developed a systematic methodology to detect near real-time performance changes during EHR implementations using a large set of measures identified in the literature [7] and suggested by subject-matter experts [8]. The method was tested in a large commercial EHR implementation involving 4 medium-size hospitals and 39 clinics from a large care delivery system [9]. While the proposed methodology was able to effectively detect *what* and to *what extent* changes happened, it was not designed to elucidate the dynamics surrounding *how* they happened. The objective of the present study is to identify factors that may have contributed to changes detected on quality, productivity, and safety outcomes during a large commercial EHR implementation in order to increase our understanding of the full impact of HIT interventions and to guide future research. To elicit those factors, we augmented our quantitative findings with semi-structured, in-depth interviews with clinical employees from 1 medium-size hospital and 10 clinics from the larger implementation previously monitored [9].

## Methods

### Description of the previous longitudinal evaluation

Intermountain Healthcare, a not-for-profit, integrated care delivery system of 23 hospitals and over 185 clinics covering Utah and southern Idaho, has completed the replacement of a set of homegrown legacy systems [10,11] with the commercial Millennium EHR (Cerner Corporation, Kansas City, MO, USA). The Cerner EHR implementation used a phased approach with the introduction of the new EHR across 10 geographical regions at different points in time. The implementation in each region followed a “big bang” strategy, replacing all legacy systems at once within that region. We have conducted a longitudinal evaluation of the implementation in the first five regions using an interrupted time-series design with parallel control sites [9]. We collected monthly data from February 2013 to July 2017 for 41 outcomes including quality (11 measures), productivity (20 measures), and safety (10 measures), selected from an inventory of outcomes likely impacted by HIT interventions [8]. Data needed to calculate the outcomes were collected from Intermountain’s enterprise data warehouse (EDW) containing EHR data. Data became available in the EDW after the EDW team ensured that there were no systematic differences in the data generated by the EHRs (legacy vs. Cerner). Data were analyzed using an ordinary least squares model [12] that assessed whether the outcomes monitored were impacted immediately after the introduction of the implementation (i.e., EHR “go live”) and compared the average change per month in the outcome before and after the go live. Table 1 lists the quantitative outcomes from our previous evaluation [9] that represent the quantitative phase of the present mixed-methods investigation.

**Table 1:** Outcome measures from the longitudinal study included in the qualitative analysis.

Type of measurement	Measure	Description	Significant impact observed at the respondents’ settings

Primary care quality measures	Blood pressure control	Rate of diabetes patients with blood pressure in control	Decreased immediately after the go live with no recovery to the baseline level
	Diabetes bundle	Composite measure for rate of diabetes control	Decreased immediately after the go live with no recovery to the baseline level
Primary care productivity measures	Laboratory orders	Number of laboratory test orders	Decreased immediately after the go live with no recovery to the baseline level
	New patient visits	Rate of new patient visits to ambulatory settings	Decreased immediately after the go live with no recovery to the baseline level
	Patient visits	Number of patient visits to ambulatory settings	Decreased immediately after the go live followed by a recovery to the baseline level within 11 months
	Time documenting after hours	Time spent by provider documenting in electronic health records after 6 p.m.	Increased per month after the go live*
Hospital quality measure	Readmission rate	Rate of heart failure patients readmitted within 30 days	Decreased immediately after the go live with no recovery to the baseline level
Hospital productivity measures	ED LOS	Length of stay of patients in the emergency department	Increased immediately after the go live followed by a recovery to the baseline level within 12 months
	ED visits	Number of patient visits to the emergency department	Decreased immediately after the go live followed by a recovery to the baseline level within 1 month
	ED wait time	Mean time between patient arrival and seen by provider in the emergency department	Increased immediately after the go live followed by a recovery to the baseline level within 6 months
	Employee turnover	Rate of employee contracts terminated	Increased immediately after the go live followed by a recovery to the baseline level within 12 months
Hospital safety measures	Abdominal hysterectomy infection rate	Rate of hospital-acquired surgical site infections for abdominal hysterectomy	Increased per month after the go live with no recovery to the baseline level
	Colon surgery infection rate	Rate of hospital-acquired surgical site infections for colon surgeries	Increased per month after the go live followed by a recovery to the baseline level within 6 months
	Hospital-acquired CDiff infection rate	Rate of hospital-acquired infections of Clostridium Difficile	Decreased per month after the go live with no recovery to the baseline level
	Hospital-acquired infection MRSA rate	Rate of hospital-acquired infections of Methicillin-resistant Staphylococcus aureus	Decreased immediately after the go live followed by a recovery to the baseline level within 10 months

Abbreviations: LOS: length of stay; EHR: electronic health records; ED: emergency department; CDiff: Clostridium Difficile; MRSA: Methicillin-resistant Staphylococcus aureus. * Time documenting after hours was assessed without baseline data for comparison.

### Design and settings

We conducted a mixed-methods study with a sequential explanatory design [13]. The design integrates interpretation of the quantitative results of the longitudinal evaluation previously reported [9] with in-depth, semi-structured interviews with clinical leaders and staff from 1 hospital (375 beds) and 10 primary care clinics from one of the most recent implementation regions (region 4) [9]. We selected this region to prevent recall bias and at the same time give enough time for participants to be exposed to the new system. In this region, data were collected monthly for two years before the go live (April 2016), followed by a 16-month post-intervention period that ended when control sites went live (July 2017). Intermountain Healthcare’s Institutional Review Board approved the study.

### Procedure

We selected all outcomes from the previous quantitative evaluation that detected a statistically significant change after the implementation in the targeted settings [9] (Table 1), and invited clinical leaders from the departments that represent these outcomes (e.g., for emergency department (ED) measures we invited ED leaders) to participate in an in-depth, semi structured interview. The goal of the interviews was to identify factors that may have contributed to changes detected on the outcomes in question. The interviews were divided into three steps that lasted from 30 to 60 minutes:

1. presentation of outcomes from the quantitative study (see example in Figure 1 below and other included measures in the Supplemental Content, Figures S1 to S15);
2. open-ended questions to elicit potential contributing factors and covariates (see Supplemental Content, Table S1); and
3. request for referral to other interviewees.

**Figure 1:**
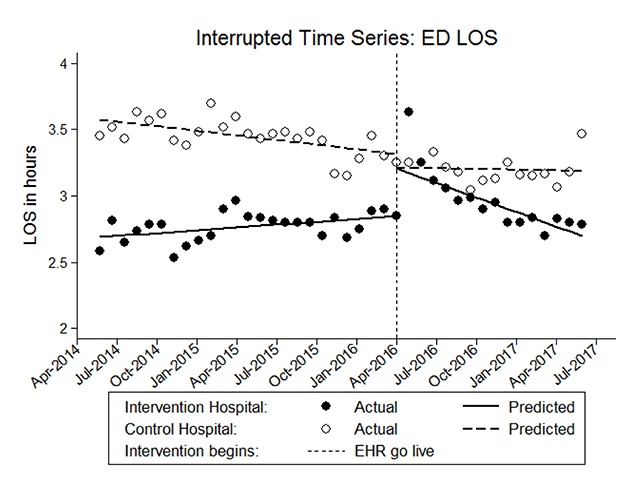
Example graph illustrating monthly length of stay in the ED with a significant increase at the intervention hospital immediately after the go live followed by recovery to the baseline within 12 months.

In the first step, we provided a brief explanation of the overall objective of the interview to ensure informants conceptualize “factors” consistently (i.e., changes to processes, procedures, or resources that could have affected – positively or negatively – the outcomes discussed). Then we presented graphical data from the quantitative study to stimulate the discussion. In the second step, once potential factors were identified, informants were asked to suggest data available in electronic format that could serve as covariates in future similar evaluations. Interviews were conducted in person from July 31, 2017, to September 29, 2017. Interviews were conducted until we were not offered any new referents to interview. We attempted to interview at least two employees for each measure in order to obtain different perspectives on the same outcome/process.

### Data Analysis

We conducted a systematic content analysis of the interview narratives based on Srnka et al.’s guidelines for analyzing qualitative data to derive new theory [14]. The analysis was conducted in 6 stages:

Stages 1 and 2: *Recording and transcription*. The audio recordings from the interviews were transcribed and de-identified.
Stage 3: *Unitization*. Transcriptions were split into units that represent informants’ responses about each outcome discussed.
Stage 4: *Coding of contributing factors*. Three authors (TKC, DB, VDC) with distinct backgrounds (informatics, medicine, nursing) independently coded relevant responses that suggest potential causes of the changes on each outcome. We initially attempted to use a combined deductive-inductive approach as suggested by Srnka et al. [14], with the sociotechnical dimensions of HIT impact proposed by Sittig and Singh [15], but found that they did not provide enough granularity and depth of the potential factors reported by the informants. We then adopted an inductive approach with each coding author independently identifying categories that explain the changes for each outcome. Multiple coding sessions were conducted. In each session, the authors collaboratively reviewed initial codes and merged them into a redefined category through consensus. The resulting codes were used in the subsequent iterations. Once all transcripts were coded, similar categories were merged based on consensus producing a final list of factors that may have contributed to the changes on each outcome.
Stage 5: *Coding of covariates*. The same steps in Stage 4 were followed for the identification of covariates to quantitatively monitor the contributing factors identified.
Stage 6: *Identification of factors related to new EHR implementation*. Once factors were identified using the coding scheme in Stage 4, the three coding authors had a final session to collaboratively reach consensus about the classification of the factors according to their relationship with the EHR implementation: *directly related, partially related*, and *not related*.

Coding of the investigation narratives was done with Atlas.ti Version 8.0.

## Results

We interviewed 14 clinical leaders and staff who reported 14 factors that they feel may have contributed to the changes detected on the outcomes. Overall, all informants confirmed that the quantitative results presented matched their perceptions regarding the effect of the implementation. Table 2 summarizes interviewees’ characteristics and Table 3 lists the factors identified. A description of each factor is given below along with example verbatim interview quotations. We also identified 17 covariates with data available in electronic format to quantitatively measure 12 of the 14 factors identified (Table 4).

**Table 2:** Informants’ characteristics.

Intermountain leaders and staff interviewed	

**Age, years (SD)**	44.2 (11.0)
**Female, n (%)**	12 (40)
Role, n (%)*	
Director	2 (14.2)
Manager	4 (28.5)
Physician	3 (21.4)
Staff	5 (35.7)
Consultant	1 (7.1)
**Department, n (%)**	
ICU	5 (35.7)
Primary Care	3 (21.4)
Emergency Department	2 (14.2)
Cardiovascular	2 (14.2)
Infection Prevention	2 (14.2)
**Main educational background, n (%)**	
Nursing	11 (78.5)
Medicine	3 (21.4)
**Current field experience, mean years (SD)**	16.0 (11.2)
**Experience with EHRs, mean years (SD)**	14.7 (6.4)
**Time working at IH, mean years (SD)**	15.4 (10.1)

* Number and percentage for role exceed 14 and 100% respectively because some interviewees had more than one role. Abbreviations: SD: standard deviation; ICU: intensive care unit; EHR: electronic health records; IH: Intermountain Healthcare.

**Table 3:** Factors that potentially affected the outcomes.

Contributing factor	Implementation-related	Outcome(s) impacted	Explanations

Decrease in communication	Yes	ED LOS^ir^ ED wait time^ir^	Due to CPOE adoption, communication between providers decreased and interruptions increased
Incomplete data migration	Yes	Laboratory orders^d^	Partial data were migrated from the legacy systems to the new EHR compromising accuracy of overdue test alerts
Increase in staff	Yes	ED LOS^ir^ ED wait time^ir^ Patient visits^dr^	12 ED nurses were hired prior to the go live Some PC physicians employed scribes to facilitate clinical documentation and recovery of patient visits
Learning curve	Yes	ED LOS^ir^ ED wait time^ir^ Patient visits^dr^ New patient visits^d^	Due to new functionality to learn, efficiency decreased and recovery to baseline levels took longer than expected
Missing functionality	Yes	Blood pressure^d^	Due to missing functionality, clinicians were unable to override a temporary hypertension to consider the patient “in control”
Redistribution of staff or work	Yes	ED LOS^ir^ ED wait time^ir^ Patient visits^dr^ New patient visits^d^ Abdominal hysterectomy^i^ Colon surgery^i^	ED Physicians decreased their patient ratios for three days only Patients were oriented to arrive earlier for their visits to recovery to normal levels of patient visits Some preventive tasks were redistributed to keep up with increased SSI cases detected
Resistance to learning or using a new EHR	Yes	Employee turnover^ir^	Some clinical personnel quit to avoid learning or using a new EHR In some cases they anticipated their retirement
System configuration	Yes	Laboratory orders^d^ Time documenting after hours^i^ Abdominal hysterectomy^i^ Colon surgery^i^ MRSA infections^dr^ CDiff infections^d^	Laboratory alerts were added progressively PC providers used a mobile app to complete visit documentation The new EHR had a more robust capability for capturing potential infections, which was improved over time
Workarounds	Yes	Blood pressure^d^ Laboratory orders^d^ Time documenting after hours^i^	Physicians started using nurses’ triage measurement of BP; the lack of double-check for measurement may have led to inaccurate BP in some cases The process for collecting lab samples at the clinics was redesigned due to CPOE adoption Physicians modified their schedules and workflow practices in order to complete electronic documentation
Change in care pathways	Partially	Readmission rate^d^	Improvements to care pathways partially introduced by the EHR may have contributed to a decrease in readmissions
Intentional decrease in volume of work	Partially	Patient visits^dr^ New patient visits^d^ Laboratory orders^d^	Physicians were seeing fewer patients in order to complete electronic documentation
Health insurance changes	No	Diabetes bundle^d^ Patient visits^dr^ New patient visits^d^ Laboratory orders^d^ Time documenting after hours^i^	Patients with health savings accounts tend to avoid chronic disease management visits which hampers management of diabetes outcomes Insurance companies stopped covering the most common tests in physical exams potentially decreasing lab orders Insurance companies started to require more strict coding of procedures contributing to longer documentation times
Patient Engagement	No	Diabetes bundle^d^	Half of the bundle items depend mostly on patient engagement on treatment
Seasonal pattern	No	ED visits^ir^ ED LOS^ir^ ED wait time^ir^	The go live was postponed due to problems in previous regions and happened in a time of a slight pick

^i^ Denotes a significant increase with no recovery to the baseline level; ^d^ Denotes a significant decrease with no recovery to the baseline level; ^ir^ Denotes a significant increase with recovery to the baseline level; ^dr^ Denotes a significant decrease with recovery to the baseline level. **Abbreviations:** EHR: electronic health records; CPOE: computerized provider order entry; BP: blood pressure; PC: primary care; ED: emergency department; LOS: length of stay; HF: heart failure; CDiff: Clostridium Difficile; MRSA: Methicillin-resistant Staphylococcus aureus; SSI: surgical site infection.

**Table 4:** Covariates for monitoring factors contributing to changes on the outcomes.

Setting	Measure	Covariate(s)	Explanations

Ambulatory	Blood pressure control	Change in hypertension pharmacotherapy Acute illness	Uncontrolled patients with no pharmacotherapy changes may be false positives Acute illnesses may cause a temporary hypertension, but patient is still considered in control
	Diabetes bundle	Individual bundle items Type of health insurance	Evaluation of individual bundle items may facilitate identification of outcomes to improve Type of health insurance may be associated with chronic disease management
	Laboratory test orders	CDS alerts accepted Lab tests covered per type of visit Patient visits	Alerts of appropriate lab test may be associated with lab orders Changes in health insurance coverage may affect volume of lab orders Patient visits may be associated with lab orders
	Time documenting in EHR after hours	Risk adjustment factor Patient visits	Risk adjustment factor may be associated with electronic documentation Previous visits may be documented during work hours
	Patient visits	Time documenting previous visits Type of health insurance	Increased documentation may decrease patient visits Type of health insurance may decrease patient visits
	New patient visits	Proportion of patients per top insurance providers	Loss of patients from top insurance may decrease the number of new patients
Hospital	ED visits	Not identified during interviews	Not identified during interviews
	ED LOS ED wait time	ED visits Provider-patient ratio Go live support personnel*	More ED visits may increase LOS and wait time Provider-patient ratio may be associated with LOS and wait time More personnel for go live support may increase efficiency by shortening the learning curve
	MRSA infections CDiff infections	Patients in isolation	More patients in isolation may decrease infection rate
	Abdominal hysterectomy infections Colon surgery infections	Number of suspected infection cases according to the CDC’s NHSN	Number of potential infections captured by the EHR may help increase identification of true cases
	Employee turnover	Employee age	Employee age may be associated with resistance to learning a new EHR potentially increasing employee turnover
	Readmission rate	Appropriate use of medication for heart failure	Adherence to care pathways for heart failure may be associated with decreased readmission rate

Source: Covariates with data available in electronic format identified by the authors in the qualitative analysis. Abbreviations: CDS: clinical decision support; EHR: electronic health records; ED: emergency department; LOS: length of stay; CDiff: Clostridium Difficile; MRSA: Methicillin-resistant Staphylococcus aureus; CDC: Centers for Disease Control and Prevention; NHSN: National Healthcare Safety Network. *Potential moderator.

### Factors directly related to the EHR implementation

Nine factors related to the EHR implementation were reported by informants.

#### Decrease in communication

ED leaders reported that due to the increased time spent on electronic documentation, communication between nurses and physicians decreased and interruptions increased, potentially impacting *length of stay* (*LOS)* and *wait time*: “*Communication decreased while interruption increased, massively. Our doctors were hiding in the physician lounge*.” Informants also perceived that both the change in communication and in the outcomes were experienced, and expected. They reported that they had sent their own nurses to support previous go lives and had observed that most of the communication between physicians and nurses became heavily dependent on the EHR, decreasing clinicians’ efficiency. No specific covariate was identified for monitoring this factor.

#### Incomplete data migration

A primary care provider reported that due to a partial data migration from the legacy to the new EHR, some clinical decision support (CDS) alerts were inaccurate, potentially decreasing volume of *laboratory orders*: “*I see a lot of overdue stuff. I don’t know if it’s overdue, so it doesn’t get ordered*.” Acceptance rate of CDS alerts could be a covariate potentially affecting *laboratory orders*.

#### Increase in staff

Primary care providers hired new personnel to help with electronic documentation in order to recover the volume of *patient visits* at baseline: “*Some physicians employed scribes*.” ED leaders increased their nursing staff to mitigate problems in *LOS* and *wait time*: “*We hired 12 more nurses over the preceding months*.” No specific covariate was identified for monitoring this factor.

#### Learning curve

The need to allow time for clinicians to be fluent with the new system hampered their efficiency in the ED potentially contributing to *longer stays* and *wait time*, as reported by an ED manager: “*Nurses became efficient with their [legacy] program with time, so you have to give people time*.” Primary care providers also reported that their practices were less efficient, which may have affected their volume of *patient visits*: “*The issue is people are learning how to use the system. It’s not only the physician. It’s also the front desk and nursing staff*.” According to informants, both the increase in ED measures and decrease in patient volume were clearly perceived during the implementation. We asked participants if they believed that lack of training and/or go live support contributed to these changes. They informed that despite having appropriate training resources available, clinicians felt that they only learned the new system *in vivo*, and that they needed more support from “technology champions”: “*Those resources have been deployed to help with go lives in other regions*.” The number of people allocated for go live support can be a covariate and/or a moderator since it may hamper clinicians’ efficiency after the go live, potentially contributing to longer *LOS* and *wait time*, and lower volume of *visits*.

#### Missing functionality

A primary care provider reported that the new EHR missed a key functionality available in the legacy system used in cases when blood pressure was temporarily high, but did not demand treatment changes. The lack of this functionality could have contributed to an artificial decrease in *blood pressure control*: “*I don’t have clinical judgement. Now it’s just the number so if they [nurses] don’t do a blood pressure clinically perfect it’s going to be high*.” The informant suggested monitoring documentation of acute illness and changes to hypertension treatment as covariates for *blood pressure control*.

#### Redistribution of staff or work

Primary care staff started to orient patients to arrive earlier as an attempt to recover to normal levels of *patient visits*, as reported by a primary care provider: “*We call them and say, ‘You need to make sure you are 10 or 15 minutes before your appointment’*.” An infectious disease specialist reported that they had to redistribute preventive tasks in order to investigate an increased volume of potential surgical site infections (SSIs) captured by the new EHR: “*We had to send out other tasks*”, which potentially increased the number of SSIs detected. ED managers reported that a difference of clinician-patient ratio between nursing and physician staffs was the most significant factor contributing to longer *LOS* and *wait time*: “*They [physicians] didn’t change their patient ratios even though they were massively increasing their workload*.” ED Informants suggested monitoring provider-patient ratio as a covariate potentially affecting *LOS* and *wait time*.

#### Resistance to learn or use a new EHR

Intensive care unit (ICU) nurses reported multiple examples of colleagues who demonstrated a resistance to learn and use the new EHR, potentially increasing *employee turnover*: “*They said, ‘the day the system goes live, I quit’*.” This resistance was perceived as more likely to affect older employees: “*It seemed to be harder on older people*.” Management had already identified this resistance before the implementation had started. When asked if they believed the lack of training and/or go live support could have impacted this outcome, an ICU manager reported that ICU management tried to implement diverse training strategies, but were still unsuccessful: “*They didn’t want to learn a new system*.” Informants suggested tracking employee age as a covariate potentially affecting *employee turnover*.

#### System configuration

System configuration includes functionality added or modified during the implementation. A primary care provider reported that CDS alerts were progressively added to the system to decrease inappropriate *laboratory orders*: “*We actually would have alerts saying, ‘Why are you ordering this, it looks like it’s not necessary’*.” Another primary care provider reported that he frequently completed documentation after hours remotely using a new mobile application: “*What about the mobile app? Last night I couldn’t sleep so I did labs from like 1:00 to 2:00 am*.” Infectious disease specialists reported that the new EHR captured more potential SSI cases than they could investigate: “*There were just so many we finally said, ‘Hey, we’re going to look at every patient in the hospital’*.” This functionality was reconfigured to reduce false- and increase true-positives, which may have contributed to increased identification of SSIs: *“With [legacy system] we could only pull [cases] based on lab results, with [new EHR] we added more criteria to improve accuracy.”* They also reported that the new system was configured to trigger automatic orders to isolate patients every time a suspected or historical infection was documented, which increased the number of patients in isolation: “*MRSA and CDiff are going down, which makes sense with isolation increasing*.” Primary care informants suggested monitoring acceptance rate of CDS alerts as a covariate potentially affecting *laboratory orders*. Infectious disease specialists suggested monitoring the number of patients in isolation as a covariate affecting *MRSA* and *CDiff infections*, and the number of potential infections captured by the EHR as a covariate for SSIs.

#### Workarounds

Reactive workflow changes affected multiple outcomes in both types of settings. Two primary care providers reported that they were not able to recheck blood pressure in some cases and started using nurses’ triage measurements, which potentially decreased *blood pressure control rate*: “*Because the log-in process was so painful, people were not rechecking blood pressures at the time.”* Due to the implementation of computerized provider order entry (CPOE), nursing staff had to wait for physicians to enter laboratory orders before collecting laboratory samples, which may have decreased the number of *laboratory orders*: “*Now they [nurses] need us [physicians] to sign off before it gets done*.” A primary care director reported that providers were oriented to document as much as possible at the time of the visit to avoid *after hours documentation*: “*We talked to the physicians to get the documentation done at the time of the visit*.” However, in most cases physicians were not able to follow the orientation and had to redesign practice workflow and schedule: “*On Tuesday, I stop seeing patients at 11:30 and chart the ones from Monday until 5 o’clock*.” Primary care informants reported that increased volume of visits may increase documentation and suggested monitoring patient visits as a covariate for *time documenting after hours*.

### Factors partially related to the EHR implementation

Two factors partially related to the EHR implementation were reported by informants.

#### Change in care pathways

A cardiovascular director reported that changes to care pathways were already being applied before the implementation, and that the new system facilitated this process potentially decreasing the rate of readmissions for heart failure patients: “*Our team was updating our protocols to improve these data; we added [order sets] for admissions*.” Informants suggested monitoring appropriate use of medication for heart failure as a covariate for readmission rate.

#### Intentional decrease in volume of work

A primary care director reported that primary care providers were oriented to limit their schedules after the go live: “*Clinics had their schedules limited in a way that would allow us to have time to deal with the new system*.” This orientation may have decreased *patient visits* and *laboratory orders*, as reported by a primary care provider: “*You have a drop in volume, so labs would probably go down*.” Informants suggested monitoring the number of patient visits as a covariate for *laboratory orders*.

### Factors not related to the EHR implementation

Three factors not related to the EHR implementation were reported by informants.

#### Health insurance changes

Primary care providers reported that new requirements for coding procedures increased *time documenting after hours*: “*We didn’t have a focus on trying to capture every single diagnosis for Medicare before*.” One primary care provider reported that insurance companies progressively removed coverage of tests ordered in physical examinations: “*The insurance change was a push back on physicians to kind of change our behavior*”, which potentially decreased *laboratory orders*. He also reported that patients are more frequently opting for health savings accounts; such patients tend to avoid chronic disease management visits, which decreased compliance to *diabetes bundle* and *patient visits*: “*People don’t come frequently for their diabetes control because it’s out of their pocket*.” He also reported that their top health insurance lost a contract close to the go live, which may have decreased the number of *new patient visits*: “*A contract with [company name hidden] was supposed to come to us but it went to [company name hidden]*.” Informants suggested monitoring risk adjustment factor as a covariate for *time documenting after hours*; type of health insurance as a covariate for *diabetes bundle* and *patient visits*; rate of laboratory tests covered for physical exams as a covariate for *laboratory orders*; and rate of patients per top health insurance as a covariate for *new patient visits*.

#### Patient Engagement

According to one primary care provider, two *diabetes bundle* items, hemoglobin A1c and eye exam, depend on patient engagement: *“They [patients] have to go to an ophthalmologist.”* He also reported: *“He [patient] is working in two jobs, eating out constantly, so his A1c is 11 now.”* The provider suggested monitoring each bundle item in isolation.

#### Seasonal pattern

The implementation happened in a period of increased *ED visits*: “This is seasonal… it wasn’t related to the new EHR.” The increased visits may have affected *LOS* and *wait time*: *“The volume itself will affect length of stay and wait time.”* ED leaders suggested monitoring the number of ED visits as a covariate for *LOS* and *wait time*.

## Discussion

To the best of our knowledge, this is the first study to investigate factors contributing to changes on a broad set of quality, productivity, and safety outcomes during an EHR implementation guided by the results of a large longitudinal evaluation. Although previous studies attempted to identify factors contributing to changes introduced by HIT adoption, they have focused on specific functionality such as CPOE [16] or specific outcomes such as medication errors [17]. The diversity of factors identified indicates that the need for organizational adaptation to take full advantage of new technologies is as important for health care as it is for other services sectors. Our findings lend support to the need for more robust HIT evaluations that consider the impact of contributing factors.

Hospital outcomes were more consistently affected by factors related to the new EHR implementation. Several factors affected ED outcomes; however, our qualitative analysis revealed that the lack of go live support intensified and expanded clinicians’ learning curve and may be the most plausible explanation for *longer stays* and *wait time*. Although an increase in nursing staff led to decreased patient ratios, the ED was still less efficient overall because ED physicians faced a significant change moving from paper-based to electronic ordering. Such inefficiency following CPOE implementation has been extensively reported by previous studies [18,19]. Most informants reported that appropriate training resources were available, but perceived that effective learning happens only with the use of the new system in the clinical environment. They suggested that additional support from “*technology champions*” after go live was needed. This learning curve could have been controlled with proper planning of go live support and anticipation of usability problems. Although *employee turnover* has been rated by subject-matter experts as the least relevant measure for assessing EHR implementations [8], our findings indicate that employees may resist learning and using a new EHR and potentially quit their jobs or advance their retirement. Such resistance could have been anticipated on an organizational level with the use of validated instruments for measuring acceptance of new technologies [20]. Surgical site infections increased after the go live mostly due to the EHR’s increased rate of detection of potential infection cases to investigate; however, this increase in detection was observed only after the functionality was improved, which was not anticipated and happened while the system was already operational. MRSA and CDiff infections may have decreased likely due to a system configuration that prospectively increased the number of patients in isolation by requiring providers to complete isolation orders generated automatically. Primary care informants indicated that a key functionality was not available in the new EHR and felt that they lost clinical judgement to document when patients were hypertensive. For example, patients may have a temporary hypertension caused by an acute illness, which would not necessarily characterize them as “uncontrolled.” The legacy system provided a functionality to allow clinicians to bypass a blood pressure measurement and consider the patient “in control”; in the new system, such functionality was not available. Identification of *missing functionality* could have been controlled by stakeholders with enhanced involvement of end-users in the design and customization of the new EHR, as recommended by experts in the field [18], but frequently ignored in similar interventions [21].

Ambulatory outcomes were more consistently affected by factors not related to the new EHR implementation. The constant changes to insurance coverage and billing documentation may have decreased the volume of *patient visits* and *laboratory orders*, and, in spite of that, added an enormous documentation burden. Other studies found that clinical documentation in the United States exceeds by large amounts similar documentation in other developed countries, and one of the potential reasons is the extensive billing requirements in the United States [22]. In our previous study, *time documenting after hours* in the new EHR ranged from 0.8 to 2.3 hours per provider per *month* [9]. The same outcome has been reported elsewhere as 1.4 hours per provider per *weekday* [23]. Our qualitative analysis found that providers frequently blocked periods of their schedule to document previous visits during work hours, such a documentation was not captured as “after hours” by our measurements, which may explain the smaller times observed at the study sites. Although insurance changes are not controlled by stakeholders, early involvement of end-users and allocation of “technology champions” for go live support are processes that can be internally controlled and could have mitigated the documentation burden. Providers suggested that a decrease in compliance with the *diabetes bundle* is more likely to have been affected by a decrease in chronic disease management visits, which results from an increased use of health savings accounts.

Several quality and safety measures did not recover to the baseline levels observed before the implementation. This is partially explained by the fact that these measures are more frequently affected by factors not entirely under the control of stakeholders, such as patient engagement or health insurance changes; however, there are exceptions such as identification of missing functionality only after the system was operational. Productivity outcomes frequently recovered to the baseline levels within 1 to 12 months. These measures are more likely to be affected by factors controlled by stakeholders such as redistribution of tasks, workarounds, and intentional decrease in volume of work. These factors were frequently reported by informants as areas in which adjustments were made to facilitate recovery.

### Implications for future research and EHR implementations

Our findings demonstrate that several preventive actions potentially under the control of stakeholders are important to manage not only during the active phase of EHR implementations, but also continuously thereafter as a part of the organization’s quality and safety efforts. We recommend more attention to preventive actions such as allocation of “technology champions” after the go live, since users seem to learn by using the system in real clinical scenarios. We also recommend more attention to the identification of missing functionality, proactive workflow redesign, and identification of deleterious workarounds. An effective strategy to identify areas needing additional go live support, missing functionality or workflow changes, is to simulate the use of the new system in the production environment, as demonstrated elsewhere [24]. Health care leaders must try to anticipate that some employees might resist learning the new EHR and develop strategies to engage these employees as early as possible. Our findings also demonstrate that although some factors may not be under the control of stakeholders, their impact should be considered when planning a new implementation. For example, ideally, an EHR go live, should carefully consider external challenges such as coincidence with an increase in volume of patients due to seasonal variations or anticipated health insurance changes (e.g., implementation of new Medicaid billing requirements). Finally, we recommend a mixed-methods approach in future evaluations including a qualitative analysis guided by longitudinal quantitative evaluations using our previously tested methodology [8,9] and monitoring of covariates. Several covariates identified can be relatively easily added to future similar evaluations as they are mostly dependent on data available in electronic format. Examples include covariates that can potentially be controlled by providers such as volume of patients, provider-patient ratio and go live support personnel, as well as covariates that are not under the control of providers, but can be quantitatively monitored such as occurrence of acute illnesses or seasonal variation of ED visits. Such an approach is necessary to improve the capacity of health care leaders, HIT vendors, and researchers to more effectively monitor EHR implementations and hopefully increase the understanding of the full impact of HIT interventions.

### Limitations

The quantitative results presented to informants at the beginning of the interviews may have biased the participants’ explanations. We were able to interview only 14 informants from only one implementation region (one hospital and ten clinics), which may have compromised identification of other factors. Nonetheless, we interviewed at least two employees for each measure, and in some cases the only employees specialized in the outcomes in question (e.g., the only two infectious disease specialists), which may have led to the identification of the most prominent factors. In addition, the informants represent a wide range of roles and departments relevant to the measures explored.

Intermountain Healthcare has extensive informatics experience and the perceptions of its employees may differ from employees of other institutions. Some factors and impacts were specific to the EHRs used in the present study, implementation of different EHR systems may identify different factors. Our analysis was limited by the measures available in our previous evaluation. For example, safety measures were not available for primary care settings. Finally, we were not able to identify covariates for two factors reported.

## Conclusions

We conducted a mixed-methods analysis of a commercial EHR implementation, integrating the results of a previously reported quantitative evaluation with semi-structured, in-depth interviews with individuals affected by the intervention and identified 14 factors contributing to changes on care outcomes. We also identified 17 covariates for monitoring 12 of these factors. Our findings demonstrate that several factors may affect outcomes in different ways during a commercial EHR implementation and lend support for more robust evaluations that consider the impact of these factors to hopefully increase our understanding of the full impact of HIT interventions.

## Additional Files

The additional files for this article can be found as follows:

10.5334/egems.269.s1 Figure S1 to Figure S15. Graphs of measures that detected a significant impact in our previous longitudinal evaluation.

10.5334/egems.269.s2 Table S1. Interview script.
