# Supplementary material for: Looking Behind the Curtain: Identifying Factors Contributing to Changes on Care Outcomes During a Large Commercial EHR Implementation
Source: EGEMS (Wash DC). 2019 May 6;7(1):21. doi: 10.5334/egems.269 (PMC6509951; doi:10.5334/egems.269)
Supplement: Figure S1 to Figure S15. — Graphs of measures that detected a significant impact in our previous longitudinal evaluation. [file egems-7-1-269-s1.pdf]

Supplementary Content

Figure S1 to Figure S15 – Graphs of measures that detected a significant impact in our previous longitudinal evaluation

Table S1 – Interview script

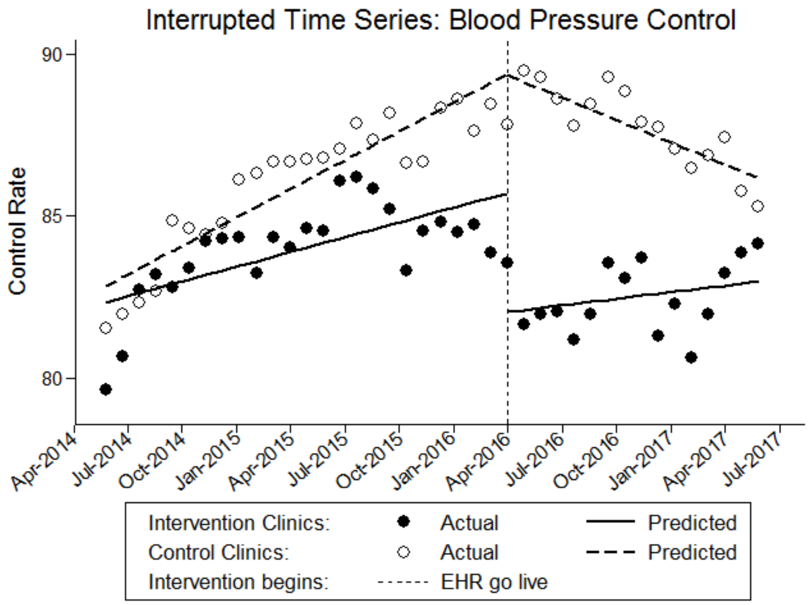

Figure S1. Blood pressure control rate per month
Source: Graph retrieved from our previous longitudinal evaluation[9]. Notes: The graph illustrates the proportion of diabetes patients with blood pressure in control over time with a significant decrease immediately after the go live followed by a significant increase over time in the intervention sites when compared to control sites.

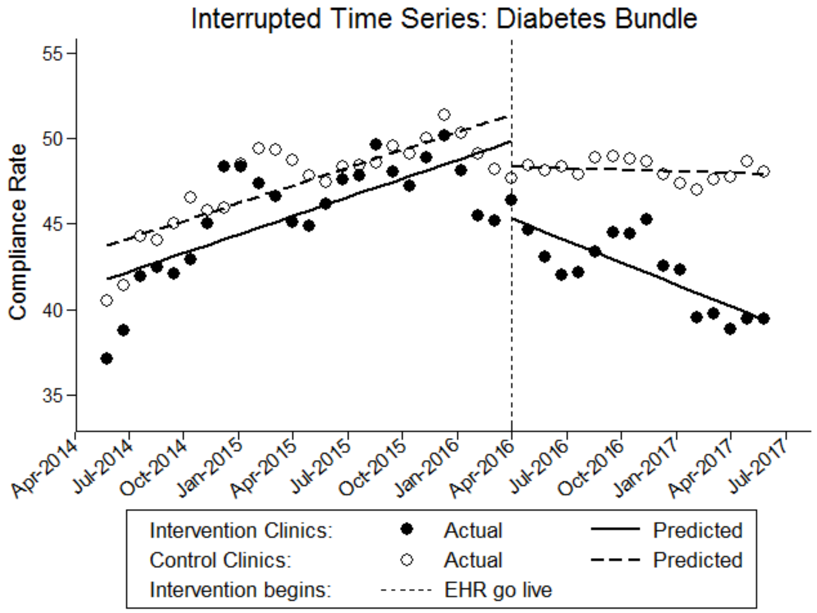

Figure S2. Diabetes bundle compliance per month
Source: Graph retrieved from our previous longitudinal evaluation[9]. Notes: The graph illustrates the proportion of diabetes patients in compliance with all bindle items over time with a significant decrease immediately after the go live and over time in the intervention sites when compared to control sites.

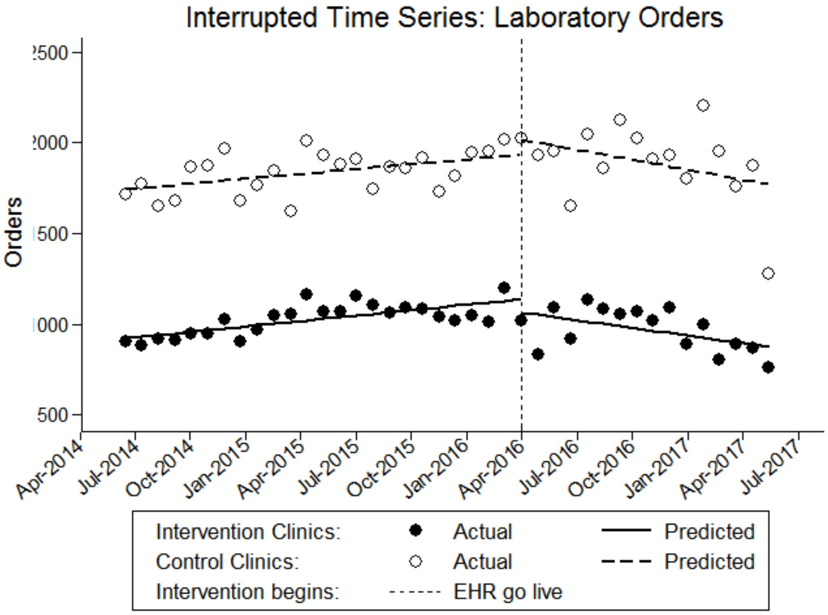

Figure S3. Number of laboratory test orders per month
Source: Graph retrieved from our previous longitudinal evaluation[9]. Notes: The graph illustrates the number of laboratory orders over time with a significant decrease immediately after the go live in the intervention sites when compared to control sites.

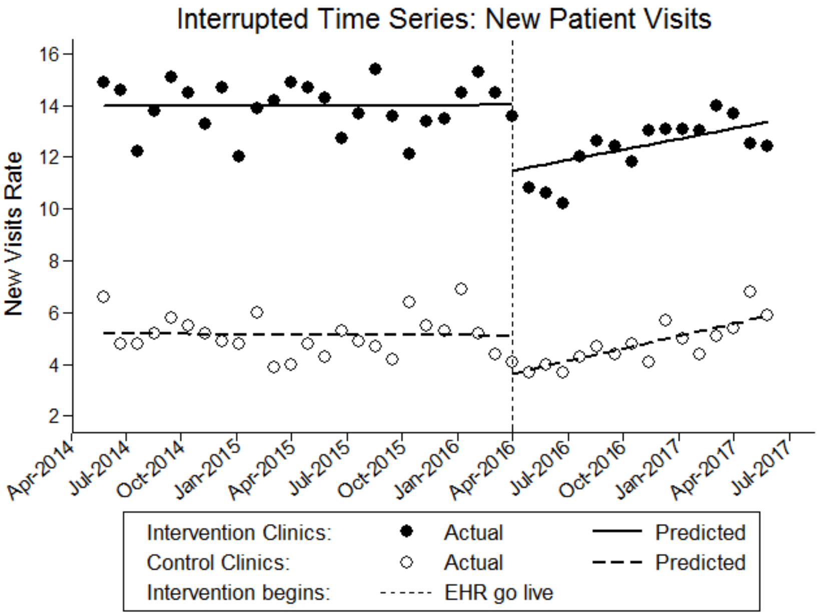

Figure S4. Rate of new patient visits per month
Source: Graph retrieved from our previous longitudinal evaluation[9]. Notes: The graph illustrates the proportion of diabetes patients with blood pressure in control over time with a significant decrease immediately after the go live in the intervention sites when compared to control sites.

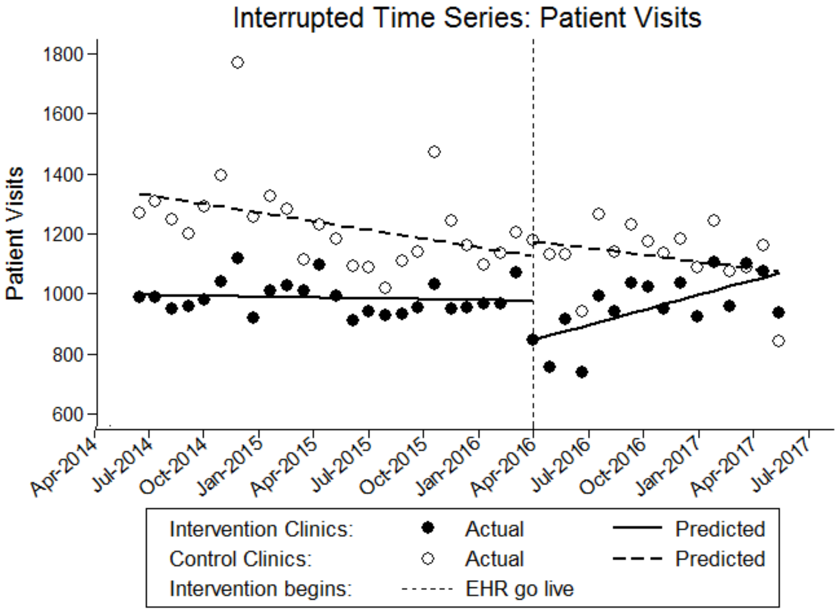

Figure S5. Total patient visits per month
Source: Graph retrieved from our previous longitudinal evaluation[9]. Notes: The graph illustrates the number of patient visits over time with a significant decrease immediately after the go live followed by a significant increase over time in the intervention sites when compared to control sites.

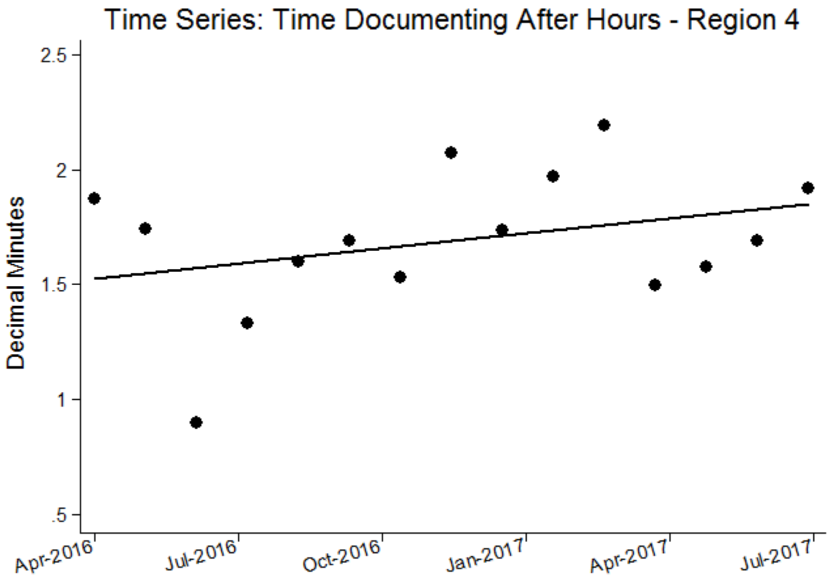

Figure S6. Time documenting in the EHR after 6 p.m. per month
Source: Graph retrieved from our previous longitudinal evaluation[9]. Notes: The graph illustrates average time documenting per provider per patient after 6 p.m. in the post-intervention period with a significant increase over time in the intervention sites. Data to calculate this measure were available only in the new EHR and were assessed in the intervention without a baseline and control.

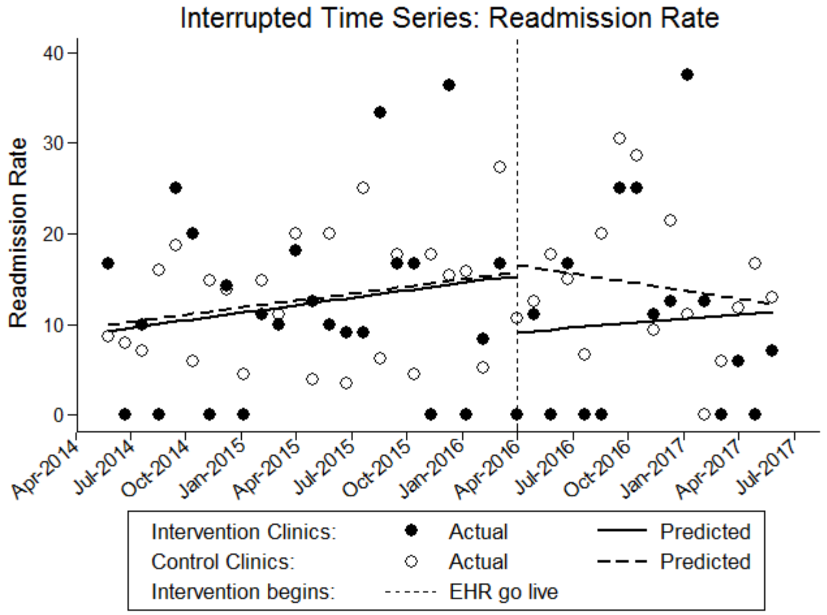

Figure S7. Rate of heart failure patients readmitted within 30 days per month
Source: Graph retrieved from our previous longitudinal evaluation[9]. Notes: The graph illustrates readmission rate over time with a significant decrease immediately after the go live in the intervention site when compared to the control site.

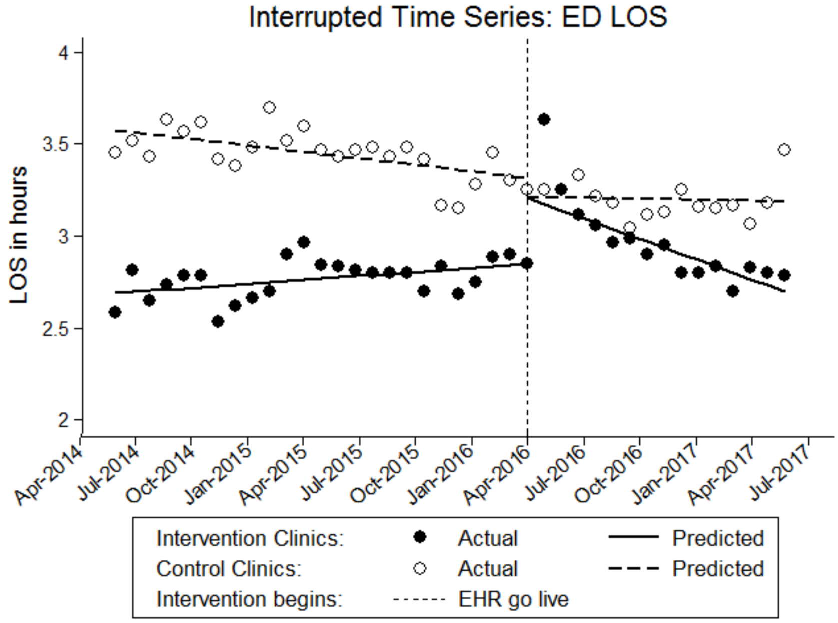

Figure S8. Emergency department length of stay per month
Source: Graph retrieved from our previous longitudinal evaluation[9]. Notes: The graph illustrates the median length of stay in hours in the Emergency Department (ED) over time with a significant increase immediately after the go live followed by a significant decrease over time in the intervention site when compared to the control site.

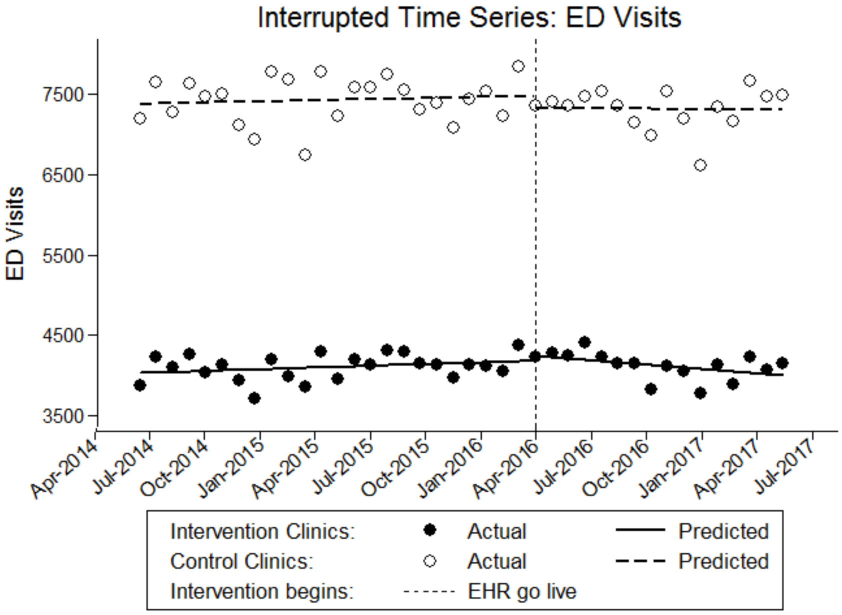

Figure S9. Emergency department visits per month
Source: Graph retrieved from our previous longitudinal evaluation[9]. Notes: The graph illustrates the number ED visits over time with a significant increase immediately after the go live in the intervention site when compared to the control site.

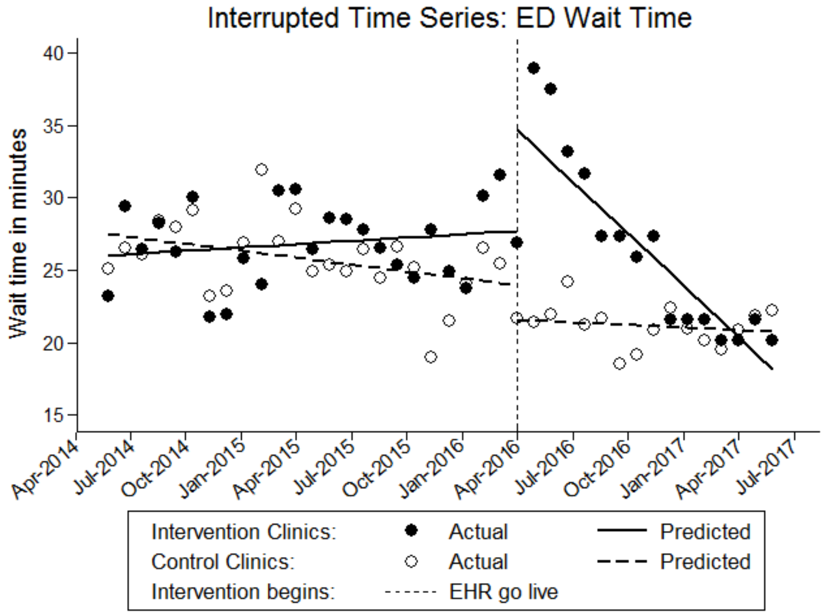

Figure S10. Emergency department wait time per month
Source: Graph retrieved from our previous longitudinal evaluation[9]. Notes: The graph illustrates the median time to be admitted in the ED over time with a significant increase immediately after the go live followed by a significant decrease over time in the intervention site when compared to the control site.

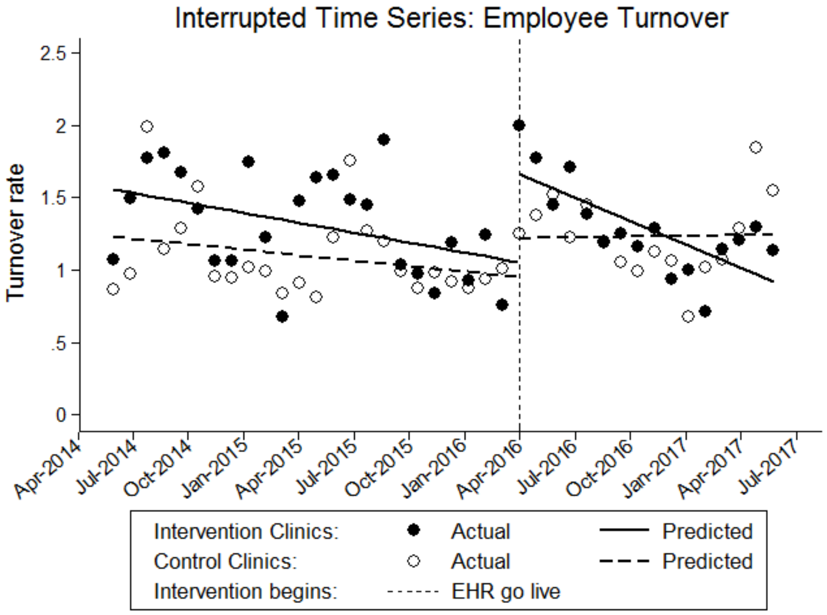

Figure S11. Employee turnover rate per month
Source: Graph retrieved from our previous longitudinal evaluation[9]. Notes: The graph illustrates the employee turnover rate over time with a significant increase immediately after the go live in the intervention site when compared to the control site.

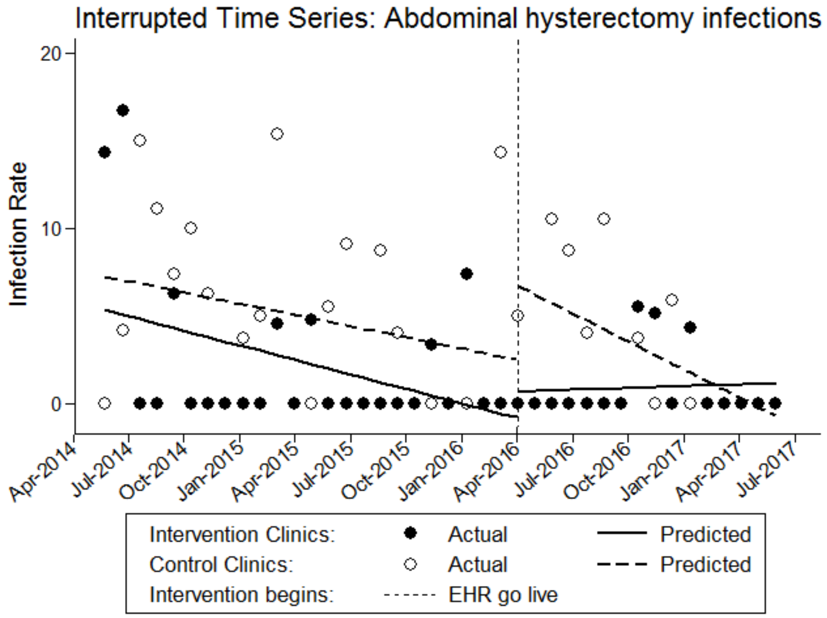

Figure S12. Rate of hospital-acquired abdominal hysterectomy infections per month
Source: Graph retrieved from our previous longitudinal evaluation[9]. Notes: The graph illustrates the rate of infections over time with a significant increase per month after the go live in the intervention site when compared to the control site.

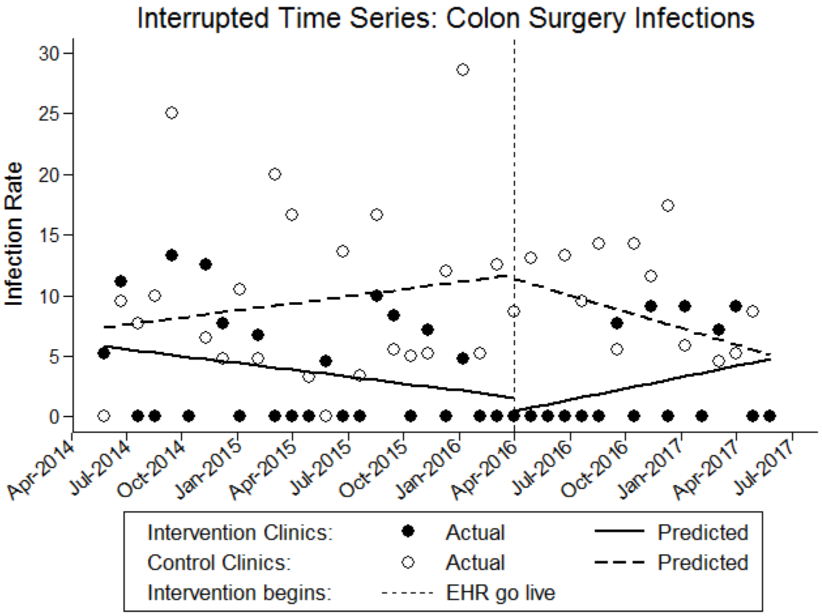

Figure S13. Rate of hospital-acquired colon surgery infections per month
Source: Graph retrieved from our previous longitudinal evaluation[9]. Notes: The graph illustrates the rate of infections over time with a significant increase per month after the go live in the intervention site when compared to the control site.

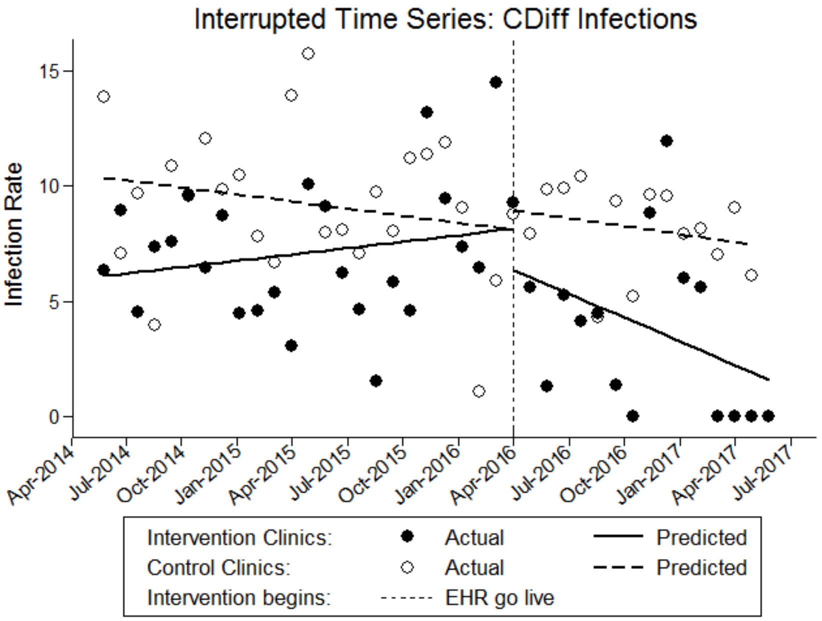

Figure S14. Rate of hospital-acquired infections of Clostridium Difficile per month
Source: Graph retrieved from our previous longitudinal evaluation[9]. Notes: The graph illustrates the rate of infections over time with a significant decrease per month after the go live in the intervention site when compared to the control site.

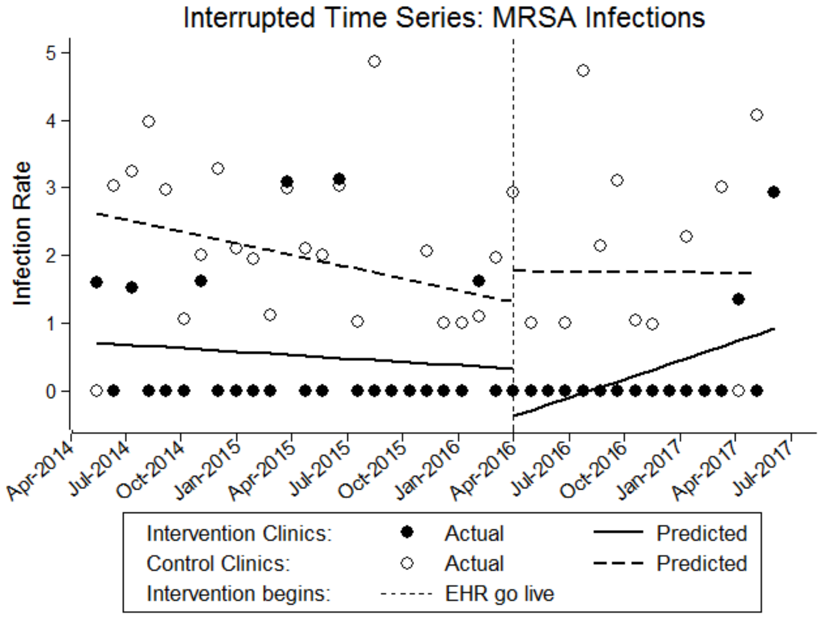

Figure S15. Rate of hospital-acquired infections of Methicillin-resistant Staphylococcus aureus per month

Source: Graph retrieved from our previous longitudinal evaluation[9]. Notes: The graph illustrates the rate of infections over time with a significant decrease immediately after the go live in the intervention site when compared to the control site.

Table S1. Interview script

Interview phase	Questions asked
Questions about perceptions of the performance changes detected and identification of complementary factors that could have caused those changes	a. During the new EHR go live, did you notice the performance change(s) on the outcomes presented here?<br><br>b. What other changes to processes, procedures, resources, and assets were introduced during the implementation of the new EHR?<br><br>c. Do you believe these changes are associated with the outcome(s) here presented? If so, how did the change(s) impact the outcome and your work?
Questions about the time when these factors were introduced	a. Please describe how you were informed about and prepared for the changes previously discussed?<br><br>b. When these changes were introduced (before, after go live)?<br><br>c. Are they still impacting your work? How?
Questions to understand how these factors affected the study outcomes	a. Please tell me about any strategies implemented by the IH leadership to mitigate/maximize the impact introduced by the implementation or by the changes previously discussed?<br><br>b. Were they effective?<br><br>c. Do you believe that lack of training and/or go live support could have contributed to these changes and impacted the outcomes?
'What-if' queries: questions to identify confounders that could be measured with data available in electronic format in future evaluations to monitor the factors elicited by the interviews	a) Are there any other process or outcome with data available in electronic format that could be measured as a confounder (potential alternative explanation to the impact observed) for monitoring the changes previously discussed?<br><br>b) Other what-if queries were identified during the interviews based on the complementary factors described by interviewee.

Source: Interview script.
