# Supplementary material for: Looking Behind the Curtain: Identifying Factors Contributing to Changes on Care Outcomes During a Large Commercial EHR Implementation
Source: EGEMS (Wash DC). 2019 May 6;7(1):21. doi: 10.5334/egems.269 (PMC6509951; doi:10.5334/egems.269)
Supplement: Table S1. — Interview script. [file egems-7-1-269-s2.pdf]

Table S1. Interview script

Interview phase	Questions asked
Questions about perceptions of the performance changes detected and identification of complementary factors that could have caused those changes	a. During the new EHR go live, did you notice the performance change(s) on the outcomes presented here?<br><br>b. What other changes to processes, procedures, resources, and assets were introduced during the implementation of the new EHR?<br><br>c. Do you believe these changes are associated with the outcome(s) here presented? If so, how did the change(s) impact the outcome and your work?
Questions about the time when these factors were introduced	a. Please describe how you were informed about and prepared for the changes previously discussed?<br><br>b. When these changes were introduced (before, after go live)?<br><br>c. Are they still impacting your work? How?
Questions to understand how these factors affected the study outcomes	a. Please tell me about any strategies implemented by the IH leadership to mitigate/maximize the impact introduced by the implementation or by the changes previously discussed?<br><br>b. Were they effective?<br><br>c. Do you believe that lack of training and/or go live support could have contributed to these changes and impacted the outcomes?
'What-if' queries: questions to identify confounders that could be measured with data available in electronic format in future evaluations to monitor the factors elicited by the interviews	a) Are there any other process or outcome with data available in electronic format that could be measured as a confounder (potential alternative explanation to the impact observed) for monitoring the changes previously discussed?<br><br>b) Other what-if queries were identified during the interviews based on the complementary factors described by interviewee.

Source: Interview script.
